# Editorial: Neuromodulation of executive circuits

**DOI:** 10.3389/fncir.2015.00058

**Published:** 2015-10-08

**Authors:** M. Victoria Puig, Allan T. Gulledge, Evelyn K. Lambe, Guillermo Gonzalez-Burgos

**Affiliations:** ^1^Integrative Pharmacology and Systems Neuroscience Research Group, Hospital del Mar Medical Research InstituteBarcelona, Spain; ^2^Department of Physiology and Neurobiology, Geisel School of Medicine at Dartmouth CollegeLebanon, NH, USA; ^3^Department of Physiology, University of TorontoToronto, ON, Canada; ^4^Translational Neuroscience Program, Department of Psychiatry, University of PittsburghPittsburgh, PA, USA

**Keywords:** neuromodulation, executive function, prefrontal cortex, striatum, cognition

The executive control of behavior involves functional interactions between the frontal cortex and other cortical and subcortical brain regions, in particular with the striatum and thalamus, via parallel fronto-striatal-thalamic loops. In all of these brain regions, neuronal excitability, and synaptic transmission are regulated by serotonergic, dopaminergic, cholinergic, adrenergic, and peptidergic neuromodulatory afferent systems that are critical for optimizing cognitive task performance. By contrast, dysfunctional neuromodulation of fronto-striatal circuits is implicated in various neuropsychiatric and neurodegenerative disorders, such as schizophrenia, depression, and Parkinson's disease. Yet, despite decades of intense investigation, it remains poorly understood how neuromodulators influence the flow of neural activity in fronto-striatal circuits to facilitate cognition. Crucial pending questions in the field include (but are not limited to): (1) How the heterogeneity of neuron subtypes and their connectivity contribute to the complexity of the underlying cellular microcircuits that are substrates of neuromodulator effects. (2) Whether the numerous receptor subtypes mediating the neuromodulator effects have cell-type specific expression patterns and effects, (3) How multiple intracellular signaling cascades mediating neuromodulator receptor effects interact in individual neurons, (4) How do neuromodulators control the strength and plasticity of synaptic inputs onto different neuron types in fronto-striatal circuits, and (5) To what extent cellular, circuit and system level effects of neuromodulators are conserved across species. This Research Topic includes 10 original research articles and seven review articles addressing the role of neuromodulation in executive function at multiple levels of analysis, ranging from the activity of single voltage-dependent ion channels to computational models of network interactions in cortex-striatum-thalamus systems.

Using cell-attached recordings of single channel and ensemble currents, Gorelova and Seamans (2015) show that dopamine (DA) D1/D5 receptors enhance persistent Na^+^ current in the soma and dendrites, but not in the axon initial segment of layer 5 pyramidal cells (L5PCs) in the rat prefrontal cortex (PFC). This finding suggests a subcellular compartment-specific regulation of excitability in PFC L5PCs. Vitrac et al. (2014) find that DA D2 family receptors also modulate L5PC activity in the mouse primary motor cortex. They report that D2 receptor activation, by either systemic or intracortical administration of the D2 agonist quinpirole, enhances the firing of putative L5PCs *in vivo*. However, Dembrow and Johnston (2014) review recent evidence suggesting that neuromodulation of PFC L5PC activity by DA, serotonin (5HT), acetylcholine (ACh), or metabotropic glutamate receptors (mGluRs) may increase or decrease the probability of L5PC firing depending on their long-distance projection targets. Consistent with this hypothesis, Stephens et al. (2014) report that 5HT, via both 5-HT 1A and 2A receptors, differentially regulates L5PC activity in the PFC based on both their long-distance projection targets and their activity state (e.g., at rest, during current-induced firing, or with simulated synaptic input).

Neuromodulators can also influence synaptic signaling and plasticity in executive circuits. Ruan et al. (2014) examined spike-timing-dependent plasticity of glutamate synaptic inputs onto L5PCs in mouse PFC and found that interactions of D1/D5 and D2 DA receptors enable Hebbian and anti-Hebbian forms of NMDA receptor dependent plasticity. In their mini review, Arroyo et al. (2014) highlight recent work using optogenetic tools to address the nicotinic ACh receptor (nAChR)-mediated effects produced by selective stimulation of cholinergic axons, including studies assessing the mechanisms underlying nAChR-mediated fast synaptic transmission in cortical circuits. Bloem et al. (2014) also review studies of cholinergic modulation in PFC, focusing on how nAChRs affect signal processing in PFC microcircuits, and proposing that ACh neuromodulation of PFC circuit function is critical for attention via ACh actions on different nAChR subtypes localized in interneurons and PCs of different cortical layers.

Puig et al. (2014), reviewing DA neuromodulation of learning and memory processes across a spectrum of animal models, including birds, rodents, humans, and non-human primates, propose a highly conserved role for DA across mammals that also evolved comparatively, albeit independently, in the avian brain. Chandler et al. (2014) review the heterogeneity of DA and norepinephrine (NE) midbrain neurons, and the specific roles of subpopulations of both DA and NE neurons in PFC-dependent cognitive tasks and in mental disorders. The review by Clark and Noudoost (2014) focuses on how DA in the PFC influences the interaction between neuronal activity in PFC and in other cortical regions in non-human primates, proposing that changes in catecholamine levels in the PFC contribute to attention and working memory function. Studying the non-human primate (marmoset) brain, Shukla et al. (2014) examined the expression of mRNAs for all of the 13 members of the 5HT receptor family, finding layer- and region-specific 5HT receptor expression in cortex and subcortical structures that suggest precise co-localization of different classes of receptors with 5HT and 5HT axons. The mini review by Miguelez et al. (2014) further explores the localization of 5HT receptor subtypes in various divisions of the basal ganglia in rodents, monkeys, and humans and discusses the physiological and behavioral effects of their manipulations in relation to the potential role of 5HT in the motor and cognitive disturbances in Parkinson's disease.

Carli and Invernizzi (2014), review the crucial role 5HT and DA play in executive function and attention, focusing on the effects of 5HT and DA receptor manipulation on behavioral disturbances produced in rodents by disrupting glutamate signaling in the PFC via local NMDA receptor antagonist administration. Using a computational network model, Morita and Kato (2014) explore the possibility that DA neurons, believed to compute reward prediction errors, convey this signal to cortico-striatal circuits in part via progressive increases of DA in the striatum that controls the decay of synaptic potentiation produced during performance of reward-associated navigation tasks. Dasgupta et al. (2014) similarly used simulations in a computational model network to test the hypothesis that, to generate goal-directed control of behavior, reward-based learning (dependent on cortex-striatum-thalamus circuits) cooperates with correlation-based learning (dependent on cerebellum-thalamus-cortex circuits). Their model suggests a crucial role for neuromodulation of thalamic function in the integration of these processes. The impact of neuromodulation in the different thalamic nuclei and associated circuits is reviewed in detail by Varela (2014), who focuses on the role of midline and intralaminar groups of thalamic nuclei that may play important and specific roles in shaping executive function. Finally, Crittenden et al. (2014) report results of experiments testing the effects of overexpression of the vesicular ACh transporter in mouse brain, to assess if enhanced ACh signaling increases catecholamine levels/release, and thus modulates amphetamine-induced stereotypical behaviors that are a relevant model of behavioral alterations by drug abuse in humans.

Together, the articles summarized above demonstrate the elegant precision with which neuromodulators target specific neural circuits and subcircuits to facilitate cognition. While details of receptor expression, signaling cascades, and effector systems remain to be fully elucidated, the work highlighted in this collection demonstrates that the functional interactions between the frontal cortex and other cortical and subcortical brain regions are exquisitely sensitive to fine tuning by local release of neuromodulators. The data reported and summarized in these articles show evidence that this tuning involves neuron subtype-specific receptor expression, as well as receptor-specific effects within certain neuronal subtypes or subcellular compartments. A challenge for future studies will be linking such neuromodulatory effects at the level of the synapse or neuron with their role in plasticity at the systems level, described in the articles investigating fronto-striatal-dependent learning and behavior in animals and computational models. Fortunately, many of the cellular and system level effects appear to be conserved across mammalian and non-mammalian species, highlighting the importance of the themes addressed in this Research Topic for understanding fronto-striatal system function and dysfunction in psychiatric and neurological brain disorders.

## Conflict of interest statement

The authors declare that the research was conducted in the absence of any commercial or financial relationships that could be construed as a potential conflict of interest.
